# The use and value of digital media for information about pregnancy and early motherhood: a focus group study

**DOI:** 10.1186/s12884-016-0971-3

**Published:** 2016-07-19

**Authors:** Deborah Lupton

**Affiliations:** News & Media Research Centre, Faculty of Arts & Design, University of Canberra, Building 9, Bruce, 2601 Australia

**Keywords:** Digital media, Information, Pregnancy, Early motherhood, Parenting, Social media, Apps, Websites, Online discussion forums

## Abstract

**Background:**

Many women in countries in the global North access digital media information sources during pregnancy and the early years of motherhood. These include websites, blogs, online discussion forums, apps and social media platforms. Little previous research has sought to investigate in detail how women use the diverse range of digital media now available to them and what types of information they value. A qualitative study using focus groups was conducted to address these issues.

**Methods:**

Four focus groups were held in Sydney, Australia, including a total of 36 women who were either pregnant or had given birth in the previous three years. The participants were asked to talk about the types of digital media they used for pregnancy and parenting purposes, why they used them and in what ways they found them useful or helpful (or not). Group discussions were transcribed and thematically analysed, identifying the dominant information characteristics identified by women as valuable and useful.

**Results:**

Nine characteristics emerged from the focus group discussions as most important to women: information that was: 1) immediate; 2) regular; 3) detailed; 4) entertaining; 5) customised; 6) practical; 7) professional; 8) reassuring; and 9) unbiased. These characteristics were valued for different purposes and needs. Digital media provided women with details when they most needed them or at times when they had opportunities to access them. The study showed that women value apps or digital platforms that are multi-functional. The findings revealed the importance of using digital information for establishing and maintaining social connections and intimate relationships with other mothers. However, participants also highly valued expert advice and expressed the desire for greater and more ready access to information and support offered by healthcare professionals.

**Conclusions:**

Pregnant women and those with young children place a high value on the information and support they receive from and sharing using online sources and apps. They are accustomed to ready and immediate access to information using digital technologies and want better access to that offered by professionals. Recognising and finding ways to meet these needs should be included in planning healthcare provision and support for this group. Further research with women from socioeconomically disadvantaged backgrounds and non-urban locations is required to identify whether they have different information needs and values from the women who were included in the study reported here.

## Background

When they have given birth, many women experience uncertainty [1, 2], ambivalence [3], anxiety [1, 4] and feelings of loneliness and social isolation [2, 5] in coping with the demands of caring for a newborn. Pregnant women and mothers of young children have used online media for emotional support, connection with other parents and information since the early years of the internet in the 1990s. At first, websites, online discussion fora and blogs were available for these purposes. Over the past decade, these digital media have been supplemented by social media sites and mobile software applications (‘apps’) [6, 7]. Recent research conducted in western countries including the USA [8], Israel and France [9], Ireland [10], the UK [11, 12] and Australia [13, 14] has shown that pregnant women and mothers of young children continue to use websites and discussion fora regularly. Blogs written by other mothers (‘mommy blogs’) also continue to provide information to pregnant women and women caring for infants, as well as offering a public form of expression for writers of these blogs about their experiences [11, 15, 16]. Other studies have identified the value of apps [13, 17, 18] and social media networking sites, particularly Facebook [19–21]. Research has shown that women who are pregnant for the first time or caring for their first infant are particularly likely to use digital media [22]. Some women find that they are unable to gain enough information or support during prenatal visits, so turn to digital media to compensate [23].

Women not only use digital media to obtain information but may also act as creators of information to which other users may then have access. One integral feature of the contemporary use of digital media that requires emphasis is that users are not passive consumers of information that they find in these media. Rather, they play an active part in creating original content, as well as engaging in practices of curating, tagging, liking, recommending, sharing and sometimes reformulating the information that they come across in these media [24, 25]. Digital media users should, therefore, be considered as co-creators of digital knowledge or as information intermediaries.

Women have many opportunities to generate digital data about pregnancy and mothering. They can contribute content to blogs, online discussion fora and social media groups. They can use other digital media to generate details about themselves and their foetuses and infants. Some apps and websites facilitate self-tracking so that women can enter details about their expected date of delivery and their biometric details such as body weight, their level of physical activity, diet, moods, cravings, drug consumption as well as medical appointments and test results. Foetuses can be tracked using kick trackers or even mini-Dopplers attached to smartphones to measure foetal heart rate [26, 27]. Many other apps are available to monitor infants’ sleeping, feeding, growth and development [28]. All of these technologies create and store personal information, some of which may be shared with others.

The actors who seek to provide information for parents in digital media are extremely diverse. To nominate simply some major content providers, they include medical practitioners, paediatricians and other healthcare professionals, companies seeking to promote their products and alternative therapists as well as other parents who provide accounts of their experiences. As a result, the quality of the information provided in digital media about pregnancy, childbirth and parenting young children is widely variable. Many studies have appeared in the medical literature outlining critiques of the validity of information offered in health and medical apps [29]. As with other digital media covering health and medical issues there is little or no regulation of what information is offered in apps and online sites directed at providing information on pregnancy and parenting [30]. In their assessment of ten popular free apps relating to maternal and child health, Scott and colleagues found significant variation in the quality of content, functionality and data security provisions [31].

Users of digital media, therefore, are confronted with making judgements about which information they should trust and act on, including details they generate about themselves using digital media. This assessment goes beyond the validity and credibility of information to deeper and often tacit assumptions about the value of information. Data created and delivered on digital media platforms and devices are products of human action and decision-making [32–34]. The ways in which this information is interpreted and used is highly contextual, based on people’s life worlds. Different groups of actors give different valances to digital information, and the ways in which they do so are based on social and cultural dimensions. For example, healthcare professionals tend to have certain expectations of what health and medical data can offer them for their professional practice. In comparison, lay people value these data in other ways, based on their meaning and usefulness for their everyday lives [35].

### Aims and objectives

Most existing research on digital media for pregnancy and motherhood does not provide a detailed analysis of what type of information in contemporary digital media is the most helpful for women and how they negotiate these sources. In a technological context in which digital media are rapidly changing in what they offer users and in which apps, platforms, websites and fora are becoming more interconnected, women are potentially using these media in new ways. This article reports the findings of a focus group study involving discussion of these issues with Sydney women who were either pregnant when the groups were conducted or had given birth to a child in the previous three years. This study is one of several forming part of an integrated research programme exploring the content [26, 27, 36] and use of digital media related to pregnancy, parenting and young children.

The focus groups were conducted following a representative online survey that was one element of this research programme. The survey was completed in late 2014 by 410 women around Australia who were also either pregnant or had children under the age of three [37]. This survey focused on app use but also included questions about other digital media. The results revealed a high use of pregnancy apps: almost three-quarters of respondents had used at least one pregnancy app while half reported using at least one parenting app. The vast majority of respondents who had ever used a pregnancy app said that they found the apps useful or helpful, particularly for providing information, monitoring foetal development and changes in their own bodies and providing reassurance. While fewer women used parenting apps, those who did also found them useful as sources of information, for helping to monitor their children’s growth and development and to provide reassurance. All respondents were asked what online media other than apps they used for pregnancy or parenting information or support. Only 9 % of respondents said that they used no other online media. The answers from the other respondents demonstrated that websites remain very popular (57 % used them for pregnancy, 66 % for parenting), followed by online discussion groups (26 % for pregnancy, 28 % for parenting) and Facebook (pregnancy 22 %, parenting 33 %).

## Methods

### Participants and procedure

The focus groups were designed to follow-up some of the issues that were raised in the survey in more depth. The focus group format, involving participants discussing issues in a group setting, allows for more detailed responses than can usually be elicited from quantitative surveys. Focus groups were chosen over one-to-one interviews to facilitate group discussion and debate. Four focus groups, each with nine participants (a total of 36 women) were held in Sydney in a central city location in May 2015. The women were recruited for the groups by a market research company based in that city that specialises in running focus groups. Women who had already volunteered to be members of the company’s research panels and who fit the selection criteria (currently pregnant or had given birth in the past three years, over 18 years of age and competent in English) were contacted by the company and invited to take part in the study. The participants were remunerated with gift cards for their participation by the company.

The group discussions were designed to explore issues around the women’s use of apps and other online media for pregnancy and parenting. The participants were asked to talk about the types of digital media they used for pregnancy and parenting purposes, why they used them and in what ways they found them useful or helpful (or not). The discussion concluded with the question: ‘If you could design the ideal digital technology for women to use for pregnancy or parenting, what would it be and what would it do?’ This question was designed to elicit the participants’ ideas both about what digital media currently offered them and also what these media were failing to deliver.

All the focus groups were conducted by an experienced moderator from the market research company, who followed the question schedule devised by the author. The author was not present at the discussion groups. Three groups included women who already had young children (a total of 27 women, some of whom were pregnant at the time of the study), and the fourth was comprised of nine women who were pregnant for the first time. Eleven participants were aged between 23 and 29, 22 were aged between 30 and 39 and three were 40 years or older. They were predominantly a highly educated group. Twenty-six women held university degree qualifications. A further seven participants had technical training, and only three had high school qualifications only. Twenty-five participants identified themselves as being of Anglo-Celtic ethnicity while the remaining eleven were of Asian, South American, Continental European and Middle-Eastern ethnicity.

### Data analysis

All the group discussions were audio-taped and transcribed by a professional transcription company. The author analysed the transcripts using inductive thematic analysis. This involved identifying recurring themes within and across each group discussion by reading and re-reading the transcripts, locating the places where the participants talked about the digital information that they accessed from online media and apps and considering how their observations could be grouped into specific characteristics of information. The analysis was conducted by the author alone (this is very common for sociological and other social scientific qualitative research). To ensure rigour in the analysis of the focus group data, the author followed several verification strategies [38]. These involved the multiple reading of the transcripts and the iterative generation of themes involving identifying themes and checking against the transcripts of all the focus groups to ensure that they were shared across participants in all the groups. The findings were compared with other analyses in the author’s research programme on digitised pregnancy and parenting (including her survey referred to earlier) to reflect on whether her interpretations were valid and insightful. Verbatim quotations from the discussions were chosen to provide support for the thematic analysis and are provided in the findings to illustrate the inferences made by the author.

### Ethical considerations

In the information provided to participants, they were given contact details for an Australian helpline for people requiring emotional support, should they feel the need for such support following the discussions.

## Results

The following discussion describes the types of digital media that the women reported using, how they used these media and the judgements in which they engaged when assessing their usefulness and credibility. Nine characteristics of digital media information were identified from the focus group discussions; namely, information that was 1) immediate; 2) regular; 3) detailed; 4) entertaining; 5) customised; 6) practical; 7) professional; 8) reassuring; and 9) unbiased.

### Immediate information

A key finding from the focus groups was the participants’ constant use of search engines – and in particular, Google – to look for information about pregnancy and parenting on the internet. The women valued the opportunity that their mobile devices afforded them to go online at any time and in any location to seek such information or ask for advice. They talked about their incessant online searching, including in the middle of the night if they were worried about an issue concerning their pregnancy or their children. Women with the care of infants, for example, discussed how they could use their smartphone to search online while feeding their infants or supervising their play. Caring and online access could take place simultaneously.

The women appreciated immediate access to information from a wide variety of sources: ‘For me who likes a lot of opinions, I can go to Google something and get 50 answers straight away, whereas I’d have to read 50 books to get the same information.’ They noted that conducting an initial online search was often how they were led to parenting websites: ‘I tend to just Google the problem and just see what comes up. And the ones that come up are Baby Center, KidsHealth Central Baby …’

The instantaneous nature of this access to information was favourably compared with the wait that women may have to talk to healthcare professionals. Participants noted that they sometimes felt that they did not want to ‘bother’ healthcare professionals with queries that might be considered trivial. In these cases, the ability to go online or consult an app could provide the women with reassurance quickly and often anonymously. They noted that ‘even if it was the middle of the night’ online sources could provide advice whereas they would be reluctant to disturb a family member, friend or healthcare professional. The view was evident in the following exchange between participants in one focus group:

Participant: When you’re freaking out – “What is this?” – you can type in quickly and you get the information straight away, rather than calling a midwife and, waiting for them to get back to you.

Participant: You can do it quite easily.

Participant: And you don’t have to pester anyone, like you don’t have to call your mum, or –

Participant: Yes.

Participant: So it’s much easier, you’re not a burden on anyone.

### Regular information

Another feature of digital media that relates to the immediacy of information was the ways in which many websites, apps and social media deliver information regularly to users, without requiring their intervention. Such information is not actively sought by users but is sent to them via such features as electronic newsletters that they may have signed up to or notifications from apps or social media updates. Many said that they enjoyed receiving information from apps and websites based on their stage of pregnancy or the age of their infants, either as weekly newsletters or as daily or weekly notifications: ‘it just pops up on your phone’, as one woman put it. As another participant noted, she enjoyed receiving the daily notifications from the pregnancy app that she used about her foetus’s development:

Each day there’s something different [on the app], either about your baby’s development or it will show you a little picture of basically kind of a representation of what the baby looks like, how much it weighs, what its length is – all those kinds of things.

Social media platforms such as Facebook, as noted by the participants, also allow for the regular delivery of this kind of regular information via the status updates that appear in their feeds. Thus, for example, women receive notifications from the local mothers’ groups that they have joined on Facebook about such events as meet-ups, items for sale or news items that members think will interest other members.

### Detailed information

As well as wanting information quickly and on-demand, the focus group participants expressed their appreciation of digital sources that provided detailed information. For example, one woman described why she liked using one particular pregnancy app:

It is really cool, so you can actually go, week 29 and have a look and see what your baby’s doing and it’ll give you like a rundown of what’s happening in there. And then it also gives you a section on what’s happening in your body, so what hormones are causing what. And then it gives you a rundown of symptoms that you might be having for that week, so it might be heartburn. [It has] everything!

Digital media that provided several forms of information were valued by the participants because they did not have to spend as much time searching for details. As discussed in one group, for example, Facebook served not only as a social media networking site which they were already using for other purposes but also as a platform they could use to search for specialist groups or pages for pregnant women or mothers:

Participant: Because you’re already on Facebook, it’s one of those apps that allows you to communicate with your friends, does messaging if you need it, calendars – like it’s just one of those …

Participant: It kind of has everything

Participant: It kind of of gets to you in all different aspects.

Participant: It’s a central hub where you can then search on that and then go out. You can search for blogs, you can search for your family and keep in contact with them. Keep in contact with your parent group and all that.

### Entertaining information

Some digital media were valued as a source of entertainment, rendering the experiences of pregnancy ‘a bit more fun’, as a participant expressed it. The information provided could be reviewed at times when women were on public transport, for example, as a means of alleviating boredom:

It’s also nice to sit on the train and you’ve got all little tips and other mother’s groups, and forums and stuff you can go on as well. And cute little articles about baby names or whatever. It’s more of a distraction.

Parenting websites, blogs and apps have become increasingly interconnected and commercialised, and the most popular social media platforms offer many opportunities for pregnancy or parenting-related entertainment and commercial activity. There were relationships between information and commercial enterprises in many of the digital media used by participants. Several women talked about using platforms such as Facebook, Pinterest, Etsy or Instagram for entertainment purposes, such as viewing baby clothes and other products for infants or pregnant women to see what styles or brands were available and features offered by products:

I actually use Instagram to follow other people and companies as well, stuff like that. Like clothes, or just different ideas that inspire you about  child development or even just dressing a child. Actually it’s the only baby app I use. I follow heaps of pregnant mums and pictures of pregnancy. And then baby clothing, you know, heaps of baby stuff. I don’t have any of the other apps for a baby.

Some women also used photo apps and platforms like Instagram to take and share images of their infants:

I have PicCollage [an app], which makes collages of photos, and Instagram which I use all the time. And then there’s a nice one that I use for my baby as well, which is Magisto. It’s like an editing thing it puts music over photos and videos, so it’s like – so I send videos to the family that are really nice of the baby.

Platforms like Pinterest and Instagram were used by some women to make decisions about their purchases for pregnancy or their children and to purchase goods online. Many parenting websites and apps also provided such information, including features such as shopping guides or checklists for pregnant women preparing for birth. Women who used these websites enjoyed the details that they were able to access while browsing, noting that these activities contributed to the excitement of expecting and prepared for a baby.

### Customised information

Participants also talked about the value they placed on the customised and personalised nature of information they received from apps and online media. The use of apps to monitor feeding and sleeping patterns of infants was popular, as they provided detailed information about individual babies’ habits. First-time mothers particularly appreciated these types of apps. According to one such mother, her breastfeeding app meant she could keep track of which breast to use and how much milk her infant was taking in:

I just could not for the life of me remember – because I was breastfeeding, left breast, right breast. I didn’t ever get really full, like completely, so I could never tell left, right, how long it was. You know, I was always wondering — is [the baby] getting enough milk? Because it was all new and you don’t know what you are doing. The app was constantly just always there and it was really easy: it was just tap on, tap off.

It is interesting to note that when they were discussing the ideal type of digital technology for pregnancy and parenting, several women noted that they would like to use apps or wearable tracking devices for themselves or their infants that would generate detailed information about them. One woman, for example, described her desire for a wearable digital technology that she could place on her baby to monitor its sleeping patterns, heart rate and body temperature. Such technologies offer users the opportunity to track their pregnancies or their children even more closely, providing unique personalised information.

Many participants also wanted to be able to use apps or other digital media that could provide localised information for them, such as groups for pregnant women or mothers with young children that they could join in their area of residence, childcare services, activities for children and hospitals and birthing centres:

Later on in pregnancy, it would be nice to have an app that would be able to tell you where all the mother’s groups are, or where the breastfeeding groups are in your area. And maybe how many people go to them, or how easy it is to get into them.

### Intimate information

Another feature of information offered by apps and online media that women discussed as valuable was its intimacy, or its capacity to contribute to social relationships by sharing personal details. This was particularly the case for online fora offered by some of the pregnancy and parenting apps, websites and social media platforms that they consulted. The participants spoke about the importance of being able to exchange details of their experiences with other mothers and seek their advice if needed.

I went and did a parenting course at hospital, and we formed a Facebook group. So that’s been really helpful afterwards. There’s been parents that have had their kids already, so you can talk to them about birth and what they’re been going through. That’s been pretty good, and you can ask questions.

Sometimes women want to discuss a topic that they consider private or sensitive and therefore perhaps not easy to raise with family members or healthcare professionals. Online fora that provide anonymity and the opportunity to discuss these kinds of issues with other mothers are valued here. A participant used the example of sexual activity during pregnancy:

I think another issue is sex – having sex since the first time when you know you’re pregnant, and all that kind of stuff, which is stuff that you might not want to talk to your mum about. And it’s good, really honest responses.

This exchange of information was a way of establishing and developing social relationships. In some cases, enduring friendships were developed in these fora, even if women did not know each other’s real names. Online media such as closed Facebook pages for local mothers’ groups could build on and extend these friendships by facilitating the organisation of face-to-face meetings. Information about the best local activities and venues for parents of young children could be exchanged on these fora, again in some instances leading to meetings at these places. Here, the practice of seeking or contributing to online information was represented as a form of connection, a way of dealing with the isolation and lack of social support to which many women referred.

Several participants said they experienced the notifications that they received from apps or websites about their foetuses or children as a way of connecting with and establishing a relationship with them. For example, several pregnant women spoke about their feelings of ‘excitement’ in monitoring how their foetuses were developing each week and that these details made the pregnancy seem more ‘real’ to them, especially in the early days of their pregnancy. As one woman noted, ‘It’s kind of nice – it gives you a nice fuzzy feeling’.

### Practical information

For many of the women, advice they received from online media helped them in practical ways to learn about pregnancy, childbirth and parenting. Several women said that they had used fertility monitoring apps to help them conceive and continued to use pregnancy and parenting apps and websites to learn about how to prepare for childbirth and feed and care for newborn infants and toddlers.

YouTube and a number of parenting websites were frequently mentioned as places where many ‘how to’ videos could be found about birth or caring for infants. These videos were made both by other parents and healthcare professionals. The women who used these sources noted that a feature that they found useful was the ability to review the information again and again, unlike when they are shown to do something in face-to-face situations. They could view such activities as feeding or nappy changing techniques or find activities to do with their children:

YouTube’s really good. You can just go in and search, and then you find the video. And then it’s a real live person saying “This is how I do it.” And so it’s quite good to watch. Because you can play it back, like several times, because you kind of feel bad when someone says “You swaddle it this way” and you’re trying to take it in. And you’re like, “Okay, I didn’t get any of that!” But with a video you can do it at your own pace and go back, and see how they do it.

### Professional information

While participants expressed appreciation for the advice that other parents could provide, their discussions of the information that they sought in apps or online revealed their desire for greater access to the advice and support of healthcare professionals or respected organisations such as the Australian Breastfeeding Association.

When they were discussing the digital media they would ideally like to have access to, many women observed that they would appreciate a technology offering a live question-and-answer online forum or video call with healthcare experts such as midwives, paediatricians or child nurses so that users could request expert advice and receive answers immediately. A discussion among participants in one focus group illustrates this feature:

Participant: It would be nice if there were an online doctor, one that you can chat to instantaneously.

Participant: Definitely!

Participant: But just a midwife or someone that’s 24/7 where you can just type in your question –

Participant: Or Skype, where you can actually chat online with them.

Here the desire for immediacy of information was again evident, coupled with women’s valuing of professional expertise.

### Reassuring information

Digital sources were used by participants to provide reassurance and support when they felt worried about issues to do with their pregnancy or their infants. For example, apps related to infant development were viewed as allowing women to understand their infants’ behaviour better and providing reassurance that their infants were not atypical or problematic if they cried a lot or that their mothering skills were deficient. Pregnancy self-tracking apps allowed women to record their health and the development of their pregnancy and compare their details to expected norms.

Online discussion fora allowed women to seek reassurance from women who had experienced similar events or problems. As two women in one focus group said of the information they accessed online when they were pregnant:

Participant: It puts your mind at ease, and you want to know that you’re normal, and everything’s normal, and you’re going to have a normal pregnancy, you know.

Participant: Like the online forums are good, because it’s nice, even though like a midwife, someone might say to you, “Oh, everyone gets a bit depressed during pregnancy,” or whatever, there’s actual evidence of people saying “I felt like this, I felt like that.”

Most participants also noted that access to online information helped them when they were feeling isolated, with no-one else to turn to for help. An immigrant to Australia whose family members were all many thousands of kilometres away commented: “I don’t have any family or anything [nearby], so I use Google to answer everything I needed. You know, “What do I do here?” Because I don’t have a mum.” In contrast, information sources that created or exacerbated rather than alleviated anxiety tended to be avoided by the women. Thus, for example, some participants discussed how they actively avoiding seeking out and viewing childbirth videos on YouTube when they were pregnant because they did not want to become overly worried about their own impending birth. As one woman commented of YouTube, ‘You don’t want to see horrible horror stories of people giving birth and stuff!’

### Unbiased information

Several participants were aware that many websites and apps for pregnancy and parenting are supported by commercial companies. For example, a few women mentioned the Huggies app. While they found this app useful, they were aware that it was sponsored by a nappy company. They noted that they needed to be wary of the information that the app provided because of its ‘hidden agenda’, as one woman put it, to market their products. Some women actively avoided the use of these types of digital media because of their commercial nature.

The women were also aware that their online searches or use of sites such as Facebook identified them to advertisers as expecting a baby or mothering young children. One woman, for example, observed that: ‘my Facebook feed has changed a lot since I started researching pregnancy and babies, so it’s a lot of baby-related advertising coming up on Facebook.’ Many women also demonstrated awareness that when they searched online, often the sites that rank first do so because they have paid Google to achieve this ranking rather than for their popularity. Compared to these kinds of websites, those online sources or apps that had government backing, such as health department websites for parents, were viewed as more trustworthy.

## Discussion

This study revealed the great importance that the focus group participants placed on online access to information about pregnancy and parenting. Women discussed the often chaotic and anxiety-provoking features of pregnancy and the early days of parenthood with a newborn and their feelings of isolation. In their accounts, information that they not only accessed from apps and online media but also generated themselves provided a means of taking control again. Digital media provided women with details when they most needed them or at times when they had opportunities to access them. Even if they were not actively contributing content, the opportunity to go online at any time and seek advice from other parents, as well as healthcare professionals, was important to these women, providing them with relief from anxiety or uncertainty. For many women, tracking their pregnancy, using smartphones and apps as baby monitors and monitoring infants’ sleeping, feeding and growth patterns with apps were viewed as ways of using or generating information that gave them peace of mind.

The focus groups also revealed the importance of use of digital information for establishing and maintaining intimate relationships. While information offered by professionals was highly valued when women had a specific health-related concern, the intimacy provided by the emotional support and connection offered by other mothers online was an important factor for many women. The types of information exchanged on platforms used by the women in this study (which include online discussion groups as well as newer social media platforms such as Facebook) are valued not only for the advice that they offer but also because they are part of the currency of friendships and social networks. They helped women feel better connected not only to their peers but also their foetuses and children.

Accessing digital information about pregnancy and parenting for many women was also a means of providing entertainment for many women. It allowed them to enjoy experiences of pregnancy or motherhood and in some cases, to share these with others. These features of digital information contribute to the often playful elements of pregnancy and motherhood, representing them as enjoyable, exciting experiences as a counter to the anxiety, isolation and uncertainty some women felt.

The study further showed that women value apps or digital platforms that are multi-functional and interact with each other. Apps and websites that offer regular notifications, videos and the opportunity to input self-tracked data, for example, fulfil many information requirements for women. It is notable that Facebook was identified as more than just a social networking site used for maintaining social contacts, but as a platform that offered access to other forms of social engagement and information that was specifically tailored to women’s needs. Not only did Facebook provide social contact and connections with other women online, but it also facilitated in-person meetings via local mothers’ group communities. The opportunity to identify these communities and arrange meetings with women in their geographical area was very important to participants who desired face-to-face as well as online social encounters.

### Limitations

The findings of this study cannot be generalised, as the recruitment strategy of the study was not random or based on sampling strategies. The use of research panel volunteers from a market research company and the provision of gift cards to participants may have influenced focus group membership in unknown ways. Participants in this study were from a distinct socioeconomic group: they were all city-dwellers, generally highly-educated and could access the internet readily. Australians are among the world leaders in smartphone ownership and regular use of the internet [39]. However, in Australia as elsewhere, disparities of access remain. Australians from rural areas or socioeconomically disadvantaged groups have less access to Wi-Fi services and digital devices. It is likely that their need for digital information sources is greater than advantaged urban populations, given less availability of support from healthcare services and lower education levels [40]. Research has demonstrated that parents from different sociocultural backgrounds discuss different parenting issues in online fora [9], suggesting that sociocultural and geographical contexts of parenting are important to their information needs. Further research with women from socioeconomically disadvantaged backgrounds and non-urban locations is required to identify whether they have different information needs and values from the type of women who were included in the study reported here.

## Conclusions

The findings outlined here provide some further and more nuanced insights into why digital media sources are valued for pregnancy and motherhood. They show that pregnant women and mothers with young children now both use information that they find in digital media and actively participate in creating this information. They engage in these practices as ways of seeking emotional support, entertainment and connection with others as well as learning about pregnancy and caring for infants and small children.

There are significant implications of these findings for healthcare and family support professionals working with pregnant women and mothers of infants and young children. Women are now willing and able to generate reams of detailed information about themselves and their foetuses or infants using digital media. They can engage in close self-tracking of their own bodies and health and that of their children. Healthcare professionals need to be equipped to discuss and negotiate these self-generated details with women as well as maintain awareness of the other sources of information they access.

Furthermore, the study showed that women in these life stages are accustomed to being able to access information online easily and at any time of the day and night using mobile media and ubiquitous internet connections. They expect and want immediate access to pregnancy and parenting information. While women ascribe high value to the practical and intimate types of information they receive from non-professionals such as other women going through the same experiences, it was evident that they also want the information that professionals who are experts in pregnancy and parenting can offer them. Indeed, the study participants strongly articulated their desire to have more ready and instantaneous access to such professional information through services such as online messaging or video services like Skype offered in real-time. Recognising and finding ways to meet these needs should be part of healthcare provision and support for this group.

## Abbreviations

Not applicable.
